# Functional Characterization of Schizophrenia-Associated Variation in *CACNA1C*

**DOI:** 10.1371/journal.pone.0157086

**Published:** 2016-06-08

**Authors:** Nicole Eckart, Qifeng Song, Rebecca Yang, Ruihua Wang, Heng Zhu, Andrew S. McCallion, Dimitrios Avramopoulos

**Affiliations:** 1 McKusick-Nathans Institute of Genetic Medicine, Johns Hopkins University, Baltimore, MD 21205, United States of America; 2 Pharmacology and Molecular Sciences, Johns Hopkins University, Baltimore, MD 21205, United States of America; 3 Psychiatry and Behavioral Sciences, Johns Hopkins Medicine, Baltimore, MD 21205, United States of America; University of Iowa Hospitals & Clinics, UNITED STATES

## Abstract

Calcium channel subunits, including *CACNA1C*, have been associated with multiple psychiatric disorders. Specifically, genome wide association studies (GWAS) have repeatedly identified the single nucleotide polymorphism (SNP) rs1006737 in intron 3 of *CACNA1C* to be strongly associated with schizophrenia and bipolar disorder. Here, we show that rs1006737 marks a quantitative trait locus for *CACNA1C* transcript levels. We test 16 SNPs in high linkage disequilibrium with rs1007637 and find one, rs4765905, consistently showing allele-dependent regulatory function in reporter assays. We find allele-specific protein binding for 13 SNPs including rs4765905. Using protein microarrays, we identify several proteins binding ≥3 SNPs, but not control sequences, suggesting possible functional interactions and combinatorial haplotype effects. Finally, using circular chromatin conformation capture, we show interaction of the disease-associated region including the 16 SNPs with the *CACNA1C* promoter and other potential regulatory regions. Our results elucidate the pathogenic relevance of one of the best-supported risk loci for schizophrenia and bipolar disorder.

## Introduction

Schizophrenia (SZ) is a complex psychiatric disorder with heritability estimated to be around 80% [1,2]. Recent genome wide association studies (GWAS) have strongly associated over 100 loci with SZ [3]. The genes imputed to these loci are highly expressed in the brain cortex and include many ion channel subunits [4]. However, in most cases the direct relationship between the variants demonstrating statistical association with disease risk and the gene they modulate remains to be established.

The single nucleotide polymorphism (SNP) rs1006737 has been associated with risk for both SZ and bipolar disorder (BP). The two diseases have a degree of overlapping symptomatology and it was long suspected that they may share genetic risk [5,6]. Recent GWAS have decisively confirmed this [3,7,8]. The SNP rs1006737 is located in intron 3 of *CACNA1C*, along with two other SNPs associated with psychiatric disease. The SNP rs2007044 is associated with SZ and has an r^2^ = 0.788 with rs1006737 [3]. The less correlated SNP, rs4765913 (r^2^ = 0.4 with rs1006737), has shown a strong association in a combined BP and SZ sample [9].

*CACNA1C* encodes the alpha subunit of an L-type voltage gated calcium channel. Calcium channel subunit genes have been implicated in multiple psychiatric disorders, including SZ and BP [10]. Calcium signaling is involved in neurotransmitter release and regulation of gene expression [11]. Disruption of these functions may play an important role in psychiatric disease.

SNPs identified by GWAS for complex diseases, such as SZ and BP, most often lie in non-coding sequences and are enriched for expression quantitative trait loci (eQTLs), suggesting that dysregulation of transcriptional control plays a role in complex disease pathogenesis [12]. The SZ and BP-associated variant in *CACNA1C* is located in intron 3, more than 100 kb from each flanking exon, and does not have any coding variants in significant linkage disequilibrium (LD). We considered, therefore, that this SNP, rs1006737, or one of the many non-coding SNPs in LD with it, might regulate *CACNA1C* expression in the brain. Differences in regulatory activity between the two alleles might cause downstream changes that modulate risk of psychiatric disease.

Here, we characterize rs1006737 and all SNPs in strong LD in four ways, with the ultimate goal of explaining the association with psychiatric disease. First, we show that rs1006737 (and therefore all SNPs in high LD) is an eQTL for *CACNA1C* expression. Second, we test all 16 SNPs in high LD for differences between alleles in driving reporter gene expression and identify one, rs4765905, that consistently shows such differences. Third, we show by electrophoretic mobility shift assays (EMSA) that many of these SNP-containing sequences bind nuclear extract proteins in an allele-specific manner, and using protein microarrays we report on specific candidate proteins. Finally, we characterize the *CACNA1C* regulatory landscape using chromatin conformation capture followed by next generation sequencing (4C-seq) and demonstrate protein-mediated interactions between the *CACNA1C* promoter and the disease-associated interval and a few other regions of potential interest.

## Results

### eQTL Analysis in Normal Brain Tissue

**Fig 1:**
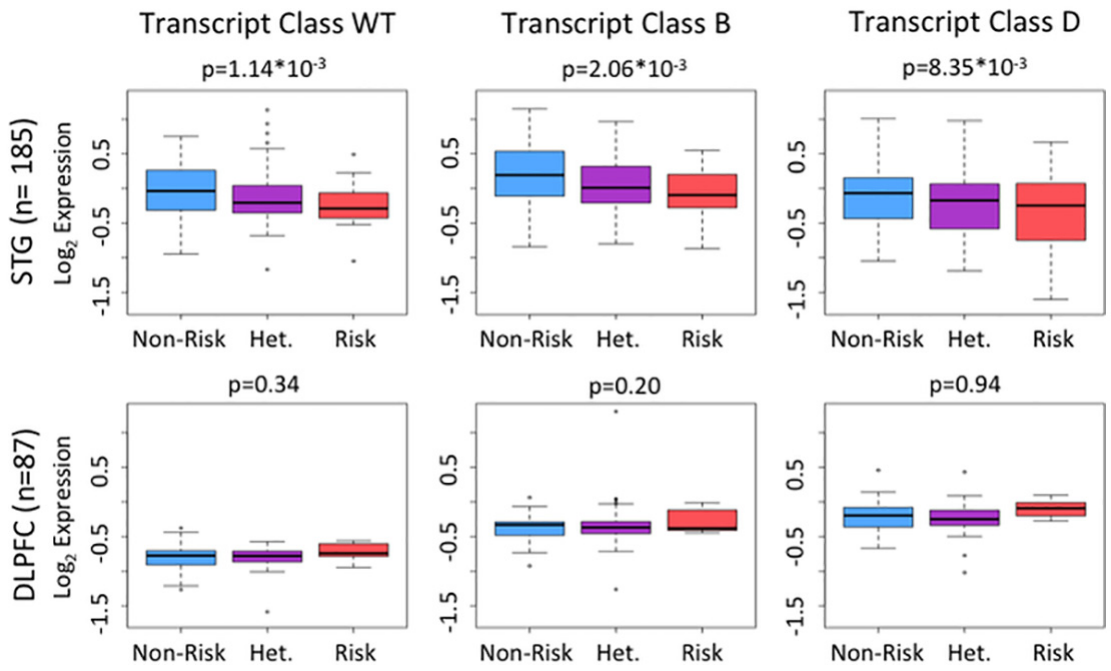
Log transformed expression of the three *CACNA1C* transcript classes. *CACNA1C* transcripts are measured by qPCR and grouped by genotype at rs1006737. Samples homozygous for the non-risk allele (GG) are shown in blue, heterozygous (GA) in purple, and homozygous for the risk allele (AA) in red. Samples from the STG in the top row, and DLPFC in the bottom row. Regression p-values for effects of genotype are shown over each graph.

We genotyped rs1006737and measured mRNA expression in 185 samples from the superior temporal gyrus (STG). This region is implicated in auditory processing, language, and social cognition [13–18] and shows reduction in grey matter volume in patients with BP [19] and first-episode SZ [20]. Given the extensive alternative splicing reported for *CACNA1C* in the UCSC genome browser (http://genome.ucsc.edu) and GenBank (www.ncbi.nlm.nih.gov/genbank), we decided to restrict our search to selected classes of transcripts. Following the designation introduced by Tang, *et al*. [21], we designed qPCR primers to assay three classes of splice variants previously shown to encode distinct *CACNA1C* isoforms that produce calcium channel subunits with different activation potentials. Transcripts where exon 32 splices to 33 are called WT class, transcripts where an alternative internal donor splice site is used in exon 32 are B class, and transcripts skipping exon 32 are D class. The primer design is shown in S1 Fig. We found that the risk allele (A) of rs1006737 was correlated with progressively decreased expression of all three classes of *CACNA1C* transcripts (WT class p = 1.14*10^−3^; B class p = 2.06*10^−3^; D class p = 8.35 *10^−3^) (Fig 1).

Bigos, *et al*. [22] had also previously reported a correlation between the risk genotype and the expression of *CACNA1C* in a study performed on the dorsolateral prefrontal cortex (DLPFC) of 269 brains, assayed using microarrays that measured the combined levels of all splice variants. That study reported an effect in the opposite direction to the one we observe in the STG. To test whether this is a region specific effect, we extended our analysis to include 87 independent samples from the DLPFC. In this smaller sample, we observed a non-significant trend for the risk allele (A) towards increased expression for all three *CACNA1C* transcripts, the same direction as Bigos, *et al*. (Fig 1). The potential pathological relevance of this region is reinforced by the observation that patients with SZ show activity deficits by fMRI in the DLPFC [23] and by its involvement in executive functions, including working memory, decision making, and organization [24].

### Enhancer Activity Assays

The disease-associated SNP rs1006737 marks a large haplotype block (~68 kb), with multiple SNPs in high LD; any one (or more) of which might be responsible for the observed disease association and the correlation with the *CACNA1C* transcript levels. To identify those functional SNPs, we tested each SNP for enhancer activity by dual luciferase reporter (DLR) assays in two different cell lines: SK-N-SH cells, which are derived from a human neuroblastoma metastasis and HEK293 cells, which despite being derived from kidney show a similar transcriptional profile to neurons [25]. We tested in total 16 SNPs for allele-specific regulatory activity. Fourteen SNPs were correlated with rs1006737 at an r^2^ of 0.8 or higher (rs7965923, rs769087, rs1006737, rs2159100, rs12315711, rs11062170, rs4765905, rs758170, rs10774035, rs10774036, rs10744560, rs12311439, rs1024582, rs4298967), while another two were included (rs34382810 and rs2370414) because of their physical proximity (<600 bp) with rs7965923 and rs10774035, respectively. Their r^2^ with rs1006737 was >0.7 (S1 Table). In order to maximally maintain the genomic context, we cloned ~1 kb intervals encompassing each SNP into a reporter construct, upstream of an SV40 promoter driving firefly luciferase expression. In four instances, two SNPs were located within 1 kb of each other, so they were assayed in the same construct (S2 Fig). As these pairs of SNPs are also in high LD and physical proximity, we tested together only the two risk alleles or the two non-risk alleles of each SNP (S2 Fig).

**Fig 2:**
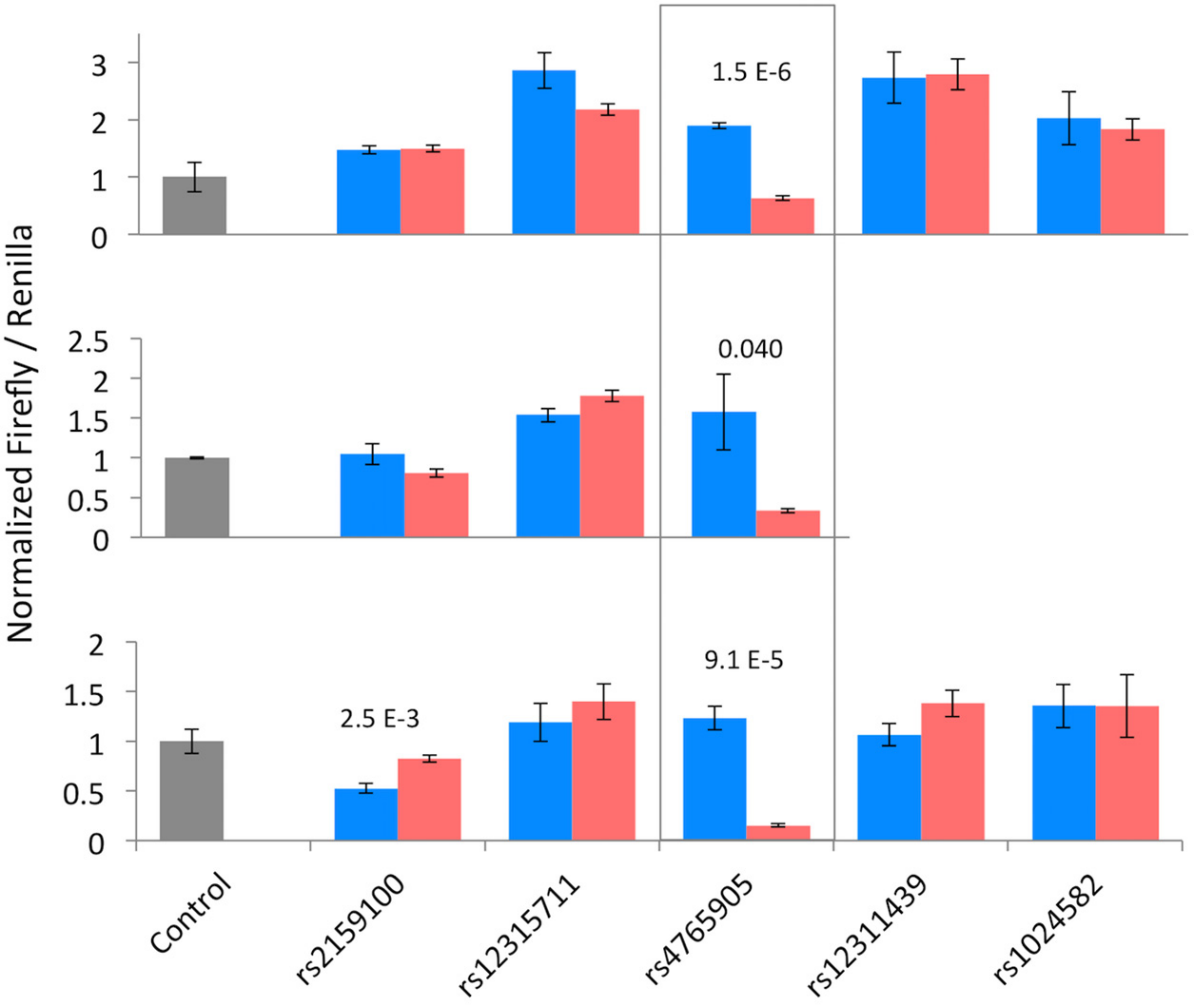
A subset of DLR constructs transfected in SK-N-SH cells. The top panel shows the same data from S2 Fig, and each successive panel shows data from replicate experiments. Relative firefly luciferase activity is shown as an average of four independent DNA extractions for each allele. Error bars represent standard error. Non-risk allele is shown in blue, risk allele is in red. Significant differences between the two alleles of a construct are indicated as p-values above the pair.

The potentially positive results for SNPs rs12315711, rs4765905, rs12311439, and rs1024582 directed us to further scrutinize these results by performing the assays multiple times in each of the two cell lines. We also included rs2159100 in the repeat assays although it showed no allele-specific difference in enhancer activity, to attempt to reproduce the findings from Roussos, *et al*. [26]. The SNPs that originally gave weaker results (rs12315711, rs12311439, and rs1024582) did not replicate in the repeat assays. SNP rs2159100 did not show any consistent allele-specific regulatory activity. SNP rs4765905, however, consistently showed significantly reduced expression of the luciferase reporter gene with the risk allele (C) in SK-N-SH cells (Fig 2). In HEK293 cells, while rs4765905 often showed significant differences, the direction was not consistent (S3 Fig).

### Protein Binding Assays

**Fig 3:**
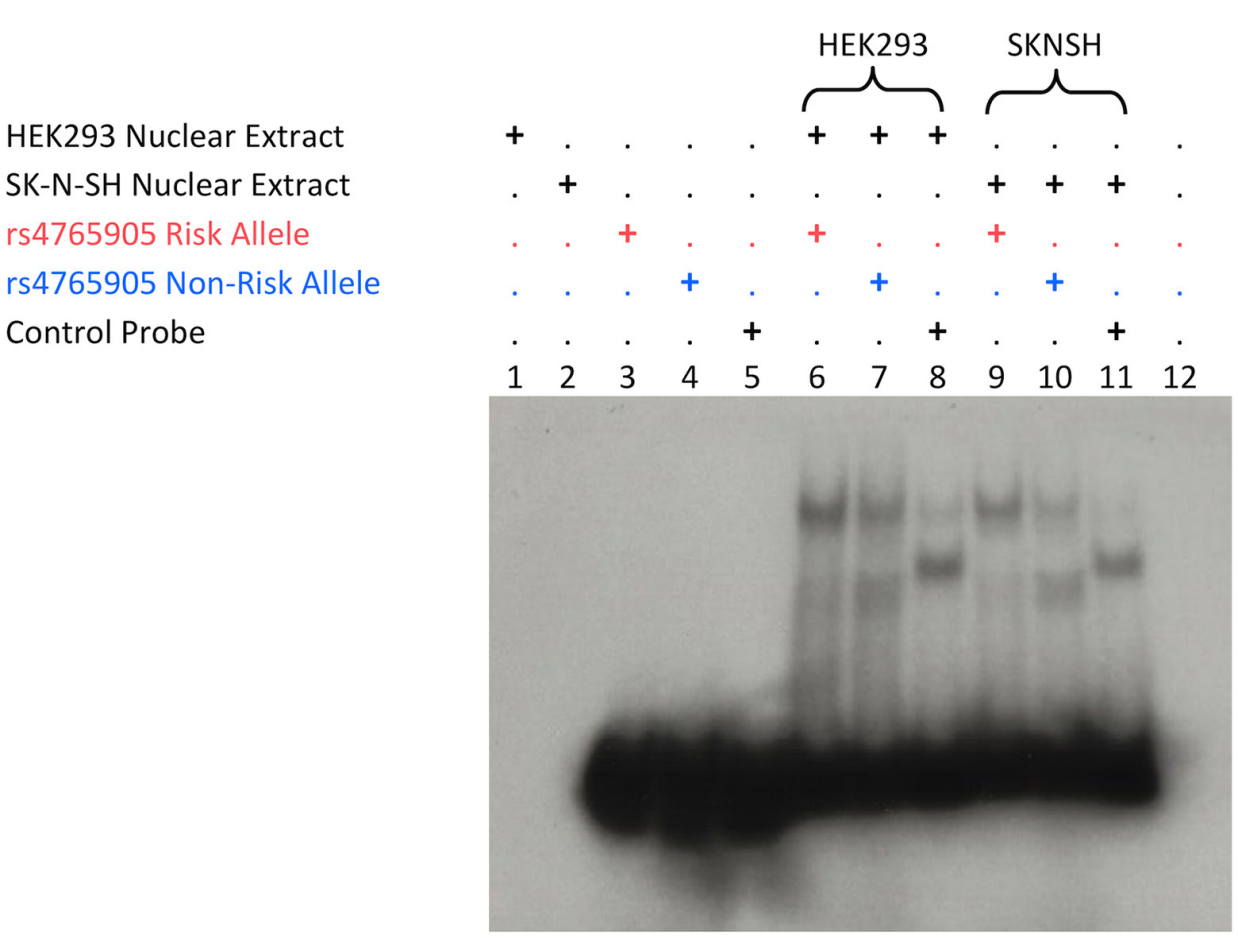
EMSA for rs4765905 with HEK293 and SK-N-SH nuclear extracts. Nuclear extracts plus buffer are run in lanes 1 and 2. Probes plus buffer are run in lanes 3–5. Probes are incubated with nuclear extract as indicated by the “+” above the lane number in lanes 6–11. Lane 12 is buffer alone. Control allele is a positive control for the assay from an unrelated variant.

To further characterize allele-specific regulatory effects, we assayed nuclear protein interactions for all 16 SNP-containing sequences by EMSA using nuclear extracts from HEK293 and SK-N-SH cell lines. We found that all sequences could bind nuclear proteins *in vitro* and that 13 of the 16 show allele-specific differences in protein binding affinity. We reproduced this and confirmed specificity of protein binding by competing out each radiolabeled allele with the reciprocal allele non-labeled (Table 1). The variant rs4765905, for which we found consistent differential enhancer effects in the DLR assay in SK-N-SH cells, showed two shift bands presumably corresponding to two different protein-binding patterns. The risk allele (C) showed stronger binding for the binding pattern migrating higher as compared to the non-risk allele (G) (Fig 3). After quantification using ImageJ [27], the ratio of top to bottom band intensity was 6.70 for the risk allele and 0.91 for the non-risk allele in HEK293 cells and 4.47 vs. 0.75 in SK-N-SH cells, respectively.

**Table 1:** Summary of EMSA results from all 16 SNPs tested.

	1	2	3	4	5	6	7	8	9	10	11	12	13	14	15	16
Protein binding	**+**	**+**	**+**	**+**	**+**	**+**	**+**	**+**	**+**	**+**	**+**	**+**	**+**	**+**	**+**	**+**
Allele differences	**-**	**-**	**+**	**+**	**+**	**-**	**+**	**+**	**+**	**+**	**+**	**+**	**+**	**+**	**+**	**+**
Competition assay	n/a	n/a	**+**	**+**	**+**	n/a	**+**	**+**	**+**	**+**	**+**	**+**	**+**	**+**	**+**	**+**

Both alleles of all 16 SNPs showed a shift, indicative of protein binding with both HEK293 and SK-N-SH nuclear extracts (top row, see S4 Fig). SNPs are in columns 2 through 17 labeled as follows: 1.rs34382810, 2.rs7965923, 3.rs769087, 4.rs1006737, 5.rs2159100, 6.rs12315711, 7.rs11062170, 8.rs4765905, 9.rs758170, 10.rs2370414, 11.rs10774035, 12.rs10774036, 13.rs10744560, 14.rs12311439, 15.rs1024582, 16.rs4298967. SNPs rs34382810, rs7965923, and rs12315711 (1,2 and 6) did not show allele-specific binding with either type of nuclear extract (middle row), and were therefore not tested with a competition EMSA (bottom row). Competition EMSAs with SK-N-SH nuclear extracts for both alleles of all SNPs, which showed consistent differences in both cell lines, confirmed the allele-specificity observed in the standard EMSAs. “n/a” indicates not assayed.

To identify the proteins capable of binding the sequences that contained these SNPs, we utilized a protein microarray containing 4,215 transcription factor and nuclear proteins [28]. Consistent with the EMSA results, all assayed sequences bound to one or more proteins. Moreover, some proteins showed allele-dependent capacity to bind (S2 Table). Interestingly, we observed that some proteins bound multiple of the assayed SNP-containing sequences. To exclude that this resulted from proteins that bind DNA promiscuously or artifacts of our array, we applied two filters. The first filter, addressing promiscuously binding proteins, was based on previously published data where we tested 460 DNA motifs on an earlier version of the array containing 1,017 proteins [29,30]. The second, addressing artifacts of the current array, was based on three negative control sequence motifs selected from those in the published experiment that did not bind any of our candidate proteins. After removing proteins binding more than six (see methods for justification) of the 460 DNA motifs (>1.3%) or any of the three negative controls, we found that five proteins binding between three and seven SNPs remained: PKNOX2, PRNP, EIF1AD, GADD45A, ZKSCAN5 (Table 2). An additional 14 proteins did not bind our negative control sequences, but were not present on the earlier version of the array, so we have no more data on their frequency of DNA binding (S2 Table).

**Table 2:** Summary of results from the protein microarray for all 16 SNPs examined.

Protein	N	1	2	3	4	5	6	7	8	9	10	11	12	13	14	15	16
PKNOX2	5	R			R	B	R	NR		R						R	
PRNP	3		NR	NR			R									NR	
EIF1AD	1							R		B						R	
GADD45A	6					NR		B				B					
ZKSCAN5	2					NR		R	R								

Proteins listed bind at least 3 of the 16 SNPs tagged by rs1006737, none of the 3 control oligos, and 6 or less of the 460 DNA motifs previously tested [29,30]. Number of DNA motifs previously bound is shown in the second column labeled N. “NR” indicates protein binds only to the non-risk allele, “R” indicates the protein binds only the risk allele, “B” indicates protein binds both alleles, blank cells indicate no binding. SNPs are in columns 3 through 18 labeled as in Table 1.

The multiple protein-binding sequences in EMSA together with the strong overlap of array-identified binding proteins may suggest that multiple SNPs participate in protein mediated complexes to regulate the expression of *CACNA1C*. This is consistent with previous reports of combinatorial haplotype effects [31].

### Circular Chromatin Conformation Capture and sequencing (4C-seq)

Based on our underlying hypothesis that *CACNA1C* is the cognate gene with which the disease-associated interval and corresponding eQTL interacts, we performed 4C-seq to describe DNA-DNA interactions within this gene. In this analysis we utilize two different viewpoints (bait sequence) as candidate cognate promoters for interactions with the disease-associated SNPs. The first was the established primary *CACNA1C* promoter. The second was the promoter of an alternative transcript previously shown to express a short part of the 3’ end of the gene resulting in a peptide called CCAT with transcription factor activity that affects the expression of the *CACNA1C* gene itself [32]. We performed experiments on SK-N-SH and HEK293 cells. To confirm initial positive results for the *CACNA1C* promoter experiments, we utilized three different restriction enzymes in independent experiments on SK-N-SH cells.

**Fig 4:**
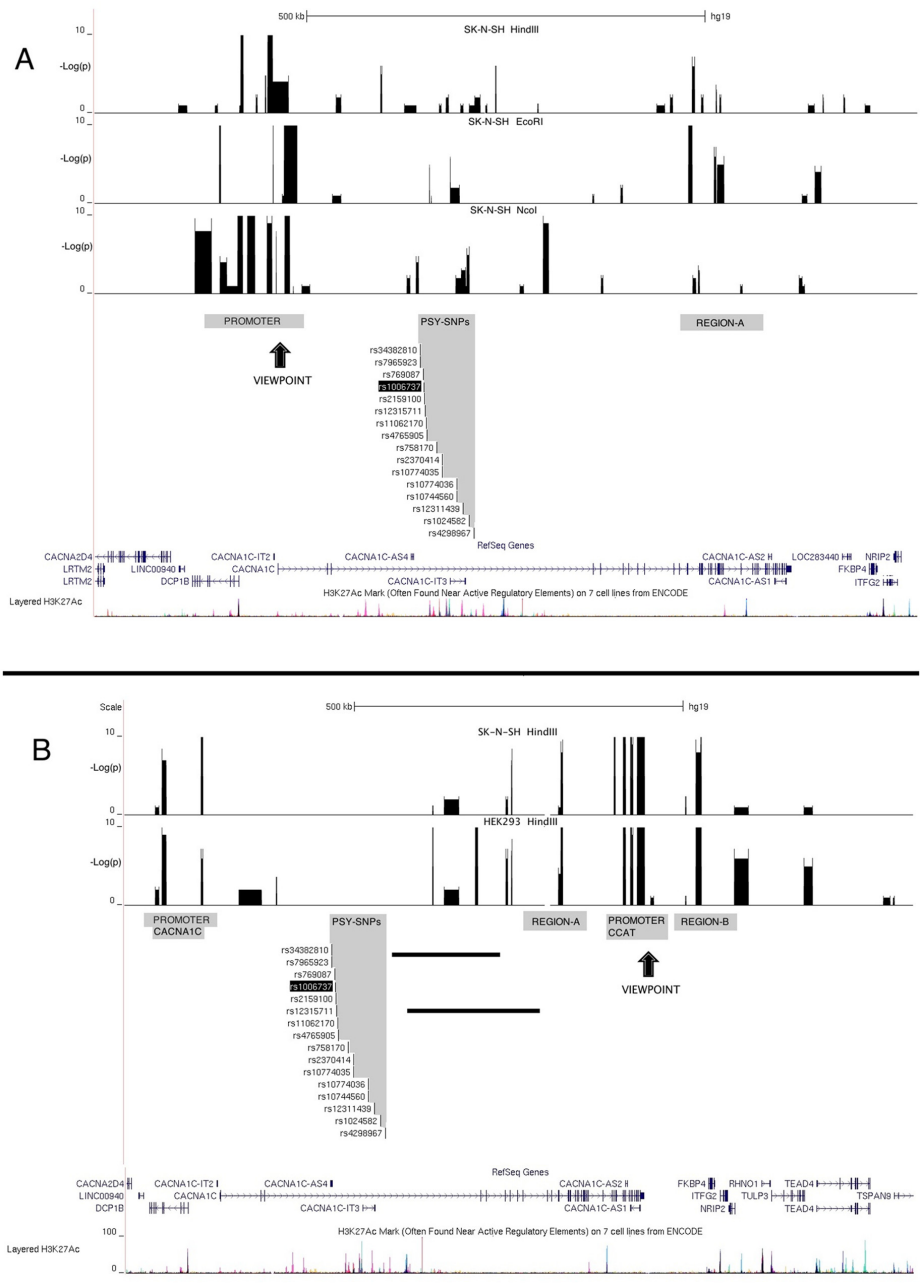
4C-seq results. (A) Results from the *CACNA1C* promoter viewpoint. (B) Results from the *CCAT* promoter. Arrows indicate the viewpoints. Bars indicate -log(p-values) for excessive read counts suggesting interaction with the viewpoint. Four regions with high densities of interactions, other than the viewpoint itself, are indicated by shading: The disease-associated SNPs in intron 3 of *CACNA1C* (labeled PSY-SNPs) and REGION A at the 3’ end of *CACNA1C* interacting with the *CACNA1C* promoter in Fig 4A, and the *CACNA1C* promoter, REGION A and REGION B downstream of *CACNA1C* interacting with the *CCAT* promoter in Fig 4B.

Fig 4A depicts the results for the *CACNA1C* promoter viewpoint in SK-N-SH cells for the three different primary enzymes (HEK293 results in S5 Fig). In addition to the region immediately surrounding the viewpoint, two additional regions consistently showed interactions with *CACNA1C* promoter. The first was the ~68 kb region in the middle of the 330 kb intron 3 that contains the disease-associated SNPs (labeled “PSY-SNPs”). The second was a region at the 3’ end of the gene beyond exon 12 (labeled “REGION A”).

Fig 4B shows results from the *CCAT* promoter viewpoint in both cell lines. This experiment did not reveal interactions with the disease-associated region and therefore was not investigated with more than one primary restriction enzyme. Consistent interaction was, however, observed in two cell lines with the *CACNA1C* promoter, REGION A, and an additional region beyond the 3’ end of the gene (labeled “REGION B”).

## Discussion

Identifying how DNA variants influence the risk for disease is a major goal in human genetics and is becoming increasingly important as GWAS start to produce large amounts of statistical associations without conclusively identifying the underlying genes and mechanisms. Our data support that the associations between rs1006737 and SZ and BP are likely due to changes in the expression of the *CACNA1C* gene. Two other SNPs in intron 3 of *CACNA1C*, rs2007044 and rs4765913, have been associated with SZ and BP, respectively. They both show at least moderate LD with our lead SNP, enhancing the evidence that this region is involved in the pathogenesis of psychiatric disease.

Here, we report that the risk allele (A) of rs1006737 is correlated with decreased expression of *CACNA1C* in the STG. In the DLPFC we observe, with a smaller sample size, a non-significant trend in the same direction as Bigos, *et al*. [22]. Although our findings in the DLPFC are consistent with a previous report by Bigos, *et al*. [22], there are significant differences. The prior study had a larger sample size, did not discriminate between transcripts, and found a significant correlation between risk allele (A) and increased *CACNA1C* expression.

Correlation between the disease-associated SNP and the gene’s expression was also reported by Rousos, *et al*. [26], using multiple public data sets, including the data of Bigos, *et al*. [22], yet with no details on strength or direction [26]. Another study of post mortem brain samples found that the risk allele (A) of rs1006737 is correlated with decreased expression of *CACNA1C* in the cerebellum, but there was no correlation in the parietal cortex [33]. Furthermore, in a study of induced neuron (iN) cells, the risk allele (A) of rs1006737 was correlated with increased expression of *CACNA1C* [34]. These results suggest there may be differences between brain regions, which may reflect the importance of finely tuned regulation of *CACNA1C* in the brain.

Our effort to identify specific variant sequences driving the association with transcript levels and disease risk produced interesting new pieces of information. First, we identify one of the 16 tested variant sequences, the sequence including rs4765905, as showing consistent allele-specific effects on driving reporter gene expression in a human neuroblastoma cell line. There are undoubtedly many reasons for false negatives in reporter assays, so we do not consider this an exclusion of the remaining sequences, but rather a reason to focus attention to rs4765905. It is quite possible that other sequences, in cis or trans, variant or not, may participate in the regulation of *CACNA1C*, perhaps even interacting with rs4765905 in the genomic context of the gene, but are not sufficient to drive gene expression alone when transfected into the cell in a reporter gene construct.

Of note, previous work by Roussos, *et al*. [26] found another SNP, rs2159100, to show allele-specific activity in reporter assays in both HEK293 and Neuro2A cells. We tested this SNP in HEK293 cells in five independent experiments but did not see the same effect. Note, however, that in our experiments the constructs contained the SV40 promoter, rather than a minimal promoter as tested by Roussous, *et al*. [26], and each of our tests involved transfecting four independent DNA preparations of each construct. The latter safeguards against false positives, especially since the DNA prep efficiency can systematically influence the results of replicates, keeping them from being true biological replicates. Either or both of these differences might explain the disagreement.

While rs4765905 showed significant allelic effects with consistent direction in SK-N-SH cells, this was not the case for HEK293 cells. Interestingly, while often showing a significant effect, the direction was not consistent. This result might once more reflect complex regulation of *CACNA1C*, similar to the differences we observed between STG and DLPFC. It is possible that unknown subtle variables affect the regulatory activity of these sequences, which in addition to the effect of the sequence variants, makes it difficult to consistently capture the differences experimentally. This might be a particularity of HEK293 cells that are known to have a heterogeneous and unstable karyotype and lack a strong tissue-specific gene expression signature [35]. This particularity might explain our discrepancies with the data of Roussos, *et al*.[26]. Nevertheless, for SK-N-SH cells, this SNP showed a consistent and significant effect over three independent experiments.

Our EMSA results support that many of the disease-associated SNPs in *CACNA1C* differentially bind nuclear proteins. Although there is no data on how often this might happen for random sequences, together with finding some on the proteins on the microarray binding multiple variant sequences puts forth the possibility that many of these SNPs may participate in protein-mediated 3D interactions and the regulation of *CACNA1C*.

Interestingly, what connects these SNPs is that they are in near complete LD, located on two segregating haplotypes associated with different *CACNA1C* mRNA levels. Such a pattern could very well be the result of balancing selection, if each regulatory haplotype gains advantage when its frequency is reduced. A similar result extending to many more examples of disease associated haplotypes has been reported by Corradin, *et al*. [31]. While all this remains a speculation, our list of proteins makes for a good starting point for further studies. The most interesting of these proteins is perhaps PKNOX2. While it binds only ~1% of the 460 previously tested DNA motifs and none of our three negative controls, it binds seven of the 16 SNP sequences, of which it shows a preference for the risk allele in five and the non-risk in one. Although PKNOX2 does not bind rs4765905, it may interact with other SNPs in the locus to fine-tune the regulatory effect of rs4765905. Interestingly, the *PKNOX2* gene, a Homeobox-Containing Gene expressed highly in the brain [36], has been previously associated with substance abuse [37] and formal thought disorder in SZ [38].

Our 4C-seq results confirmed that the region carrying the disease-associated SNPs shows interactions with the *CACNA1C* promoter. This is in agreement with the 3C experiment of Roussos, *et al*. [26] and validates the region as an eQTL. We tested interactions in HEK293 and SK-N-SH neuroblastoma cell lines, but not in brain tissue. Results from the two cell lines were very similar, yet it is not unlikely that some differences with regard to the presence or absence of interactions exist in the brain. Nevertheless, by extending from 3C to 4C, we confirm the region of interaction and find that it is not limited to the single SNP tested by Roussos, *et al*. [26], but encompasses practically all associated SNPs. Additionally, we identify a region downstream of exon 12 that also may be a regulator of the *CACNA1C* gene promoter. Interestingly, this latter region was also identified by our experiments from the *CCAT* promoter. This suggests there may be common elements in the regulation of the two genes, and it also highlights the importance of *CCAT*, which has not yet received much attention in the literature after it was first reported [32,39,40].

Our results, overall, provide strong support for a role of *CACNA1C* regulation in psychiatric disease. We show that the disease-associated SNPs in the gene are eQTLS that the reside in a region that interacts with the *CACNA1C* promoter and at least one of the SNPs can consistently drive a reporter gene in SK-N-SH cells with differences between alleles. Our protein binding experiments suggest that more than that one SNP may be involved in the complex 3D interactions that regulate *CACNA1C* expression, which is reminiscent of the proposed role of combinatorial effects of variants in LD on gene expression [31]. Such a phenomenon could be the driver of the strong LD, which links 16 SNPs in only 2 haplotypes, by favoring the allele combinations in the context of balancing selection. This phenomenon may be important for more behavioral or other phenotype associations. Better understanding of these phenomena is important towards tapping the translational potential of this and other GWAS identified associations.

## Materials and Methods

### Ethics statement

This research was approved by the Johns Hopkins University Institutional Review Board (IRB) and characterized as exempt research because all human subjects (brain sample donors) were deceased. For this reason no consent was acquired

### Gene Expression and Genotyping

#### Sample collection

Flash-frozen brain slices from the superior temporal gyrus (Broadmann area 22) of 185 donors without macroscopically visible brain pathology were provided by The Harvard Brain Tissue Resource Center. The donors had not been previously screened for the presence or absence of any psychiatric condition. They had an average age of 61.7 years, were 77.8% male, and the tissue had an average post mortem interval (PMI) from death to tissue extraction of 23.2 hours. Flash-frozen brain slices from the dorsolateral prefrontal cortex of 87 independent donors without brain pathology were acquired from the National Institute of Child Health and Human Development Brain Bank at the University of Maryland. Donors had an average age of 34.4 years, were 67.8% male, and the tissue had an average PMI of 15.0 hours.

#### DNA & RNA Extraction

Genomic DNA was extracted from 10 mg of tissue using the Gentra Puregene Tissue kit (Qiagen). RNA was extracted from 50mg of tissue using the RNeasy Lipid Tissue Mini Kit (Qiagen), and cDNA was prepared with MuLV reverse transcriptase and random hexamers (Applied Biosystems).

#### Genotyping

One SNP in *CACNA1C*, rs1006737, was genotyped with pre-designed TaqMan SNP Genotyping Assays (Applied Biosystems) using the manufacturers protocol.

*Transcript Expression*: Transcript expression levels were measured from cDNA using SYBR Green (Applied Biosystems) qPCR with transcript-specific primers overlapping exon-exon junctions. In the primer sequences below, the dash (-) represents the exon-exon junction. The primer sequences for the WT transcript are GGAATACATTTGACGCCTTGA and GGGTATGTTCAGCTGG-GTTT, for the B transcript are TGCATGGAATACATTTGACG and TATGTTCAGCTGG-CTCGG, and for the D transcript are TGATCCCTGGAATGTTTTTGA and TTGGGTATGTTCAGCTGG-ATT. The following cycling conditions were used: 95° for 10 minutes, 95° for 15 seconds, 60° for 1 minute, repeat steps 2 and 3 39 more times. A melting curve was produced and used to verify the absence of non-specific amplification or amplification of more than one sequence. Each sample had 3 technical replicates, the average of which was used for analysis.

#### Analysis

Transcript expression levels were log transformed and normalized to two housekeeping genes, *MRIP* & *ACTB* as described in Szymanski, *et al*. [41]. Correlations between genotype and normalized expression were calculated using a generalized linear model, corrected for age, sex, PMI, and plate.

### Cell Culture

HEK293 [42] and SK-N-SH [43] cell lines were obtained from ATCC. HEK293 cells were grown in Dulbecco's Modified Eagle Medium (DMEM, ThermoFisher) with 10% fetal bovine serum (FBS, Gemini Bioproducts). SK-N-SH cells were grown in DMEM with 10% FBS and 1x B27 supplement (ThermoFisher). Both cell lines were grown at 37° in 5% CO_2_.

### SNP selection

We selected for analysis 14 SNPs that were at high LD (r^2^ >0.8) with rs1006737, as identified by SNAP (www.broadinstitute.org/mpg/snap) based on 1000 genomes CEU data. Two more SNPs, rs34382810 and rs2370414, were in slightly lower LD (both r^2^ = 0.737), but were included in our constructs because of their physical proximity to SNPs in high LD. All 16 SNPs we are shown in S1 Table.

### Dual Luciferase Reporter Assays

#### Construct synthesis

Primers were designed to amplify an approximately 1kb locus including a SNP of interest (with r^2^ >0.8 with rs1006737) from genomic DNA extracted from the DLPFC brain samples with Pfu Turbo DNA polymerase (Agilent). We TA cloned amplicons into the pCR8/GW/TOPO entry vector (Invitrogen) in competent OneShot E. coli (Invitrogen). Amplicons were then recombined with Gateway LR Clonase II (Invitrogen), into a modified pGL3-promoter luciferase promoter vector (Promega), with the Gateway cassette inserted at the SmaI cut site at position 28 [44]. Plasmids were isolated with the QIAprep Spin Miniprep kit (Qiagen), digested with NotI (NEB), which cuts the destination vector once, and run on agarose gels to test their size and integrity. Inserts were Sanger sequence verified. In addition to the experimental constructs, an inert 1kb “spacer” construct was designed to control for size of insert in the vector [44].

#### Reporter Assay

Approximately 75,000 HEK293 cells or SK-N-SH cells were seeded in 24 well plates 24 hours before transfection. 4 independent DNA extracts were transfected per allele of each experimental luciferase construct. Cells were co-transfected with 0.4ug of the experimental luciferase construct and 0.04ug of Renilla transfection control (Promega) in Opti-MEM reduced serum media (ThermoFisher) using Lipofectamine 2000 (Invitrogen). After 4 hours, Opti-MEM media was replaced with DMEM media with 10% FBS. 24 hours after transfection, cells were lysed and assayed for luciferase activity with the Dual-Luciferase Reporter Assay System (Promega). Using an automated injector and plate reader system, 50uL of LARII (Promega) was added to 25uL cell lysate and the experimental luciferase reading was taken after a 3 second low speed shake and a 2 second pause. Then, 50uL Stop&Glo (Promega) was added to the sample, and the Renilla luciferase output was measured after a 3 second low speed shake and a 2 second pause.

*Analysis*: For each transfection, the ratio of experimental firefly/Renilla luciferase was calculated, and then measurements were normalized to the average of 4 biological replicates of the spacer construct. The standard error of 4 biological replicates, each replicate from the transfection of independent DNA extractions of the construct, was calculated for each allele of each SNP tested. Student t-tests were conducted to determine if the two alleles (non-risk and risk) of a given SNP showed significant differences in driving expression of the firefly luciferase reporter gene.

### Electrophoretic Mobility Shift Assays

#### Nuclear Protein Extraction

Nuclear proteins were extracted from HEK293 and SK-N-SH cells with the NE-PER Nuclear and Cytoplasmic Extraction reagents (ThermoFisher), according to the manufacturer’s protocol.

#### Probe synthesis

21 nucleotide oligos were designed centered on the SNP of interest, and both the forward and reverse complement sequences were synthesized for each allele (IDT). Forward and reverse complement oligos of the same allele of a particular SNP were added together in equal molarity and incubated at 95° for 15 minutes and allowed to cool to room temperature for 1 hour. The newly synthesized double stranded DNA was then shrimp alkaline phosphatase (SAP, Affymetrix) treated and T4 Polynucleotide Kinase (PNK, New England Biolabs) was used to add a γATP32 (Perkin Elmer) to the 5’ end. QuickClean enzyme removal resin (Clontech) was added and probes were spun in SpinX 0.22 μm cellulose acetate centrifuge tube filter columns (Costar), then EtOH purified.

#### Mobility Shift Assay

25μg of nuclear extract was incubated with 3μg BSA (New England Biolabs) and 1ug poly(dIdC, Roche) in 2x buffer (12% glycerol, 24mM HEPES, 8mM TrisHCl pH 8.0, 2mM EDTA, 1mM DTT) on ice for 10 minutes. In competition assays, non-labeled oligos were added to the reaction before the 10 minute incubation. Then 4,000 counts per million (cpm) of probe was added and incubated on ice for 20 minutes.

#### Gel Electrophoresis and Exposure

12 well TBE precast gel (BioRad) was pre-run at 150V for 20 minutes. The gel was loaded and run at 150V for 20–25 minutes, then gel was removed from the cassette, suspended between 2 sheets of cellophane, and dried. Gels were exposed to Biomax MR film (Carestream) in lead-lined cassettes at -80° and developed after 1–7 days.

### Protein Microarray

#### Probe synthesis

Probes were designed as 21 nucleotide oligos centered on the SNP of interest, with the reverse complement of the T7B primer added to the 3’ end of the oligo. Both alleles of each SNP were synthesized and hybridized to the protein array as described below. T7B forward primers with or without a Cy5 label on the 5’ end were also synthesized (IDT). T7B forward primers and the SNP probe were incubated with dNTPs, Taq, and MgCl_2_ (Invitrogen) at 55° for 5 minutes, 72° for 10 minutes, repeated for 5 cycles to generate double stranded Cy5 labeled probes or unlabeled competitors. Then probes and unlabeled competitors were EtOH purified.

#### Protein microarray

Protein microarrays were manufactured from human proteins purified from yeast GST fusion as described in Hu, *et al*. [28]. For each protein microarray, 800mL of blocking solution was prepared with 400μL 2X Base buffer (50mM HEPES pH 8.0, 100mM L-glutamic Acid Potassium Salt Monohydrate, 0.2% Triton-X, 16mM Magnesium Acetate Tetrahydrate, 20% glycerol), 2.4μL 1.0M DTT, and 400nM competitor DNA. The competitor was the opposite allele of the Cy5 labeled probe to be assayed. The protein microarray was blocked with the blocking solution using the Microarray Hybridization Chamber (Agilent) according to manufacturer recommendations for 4 hours at 4° in the dark on a nutating shaker. The gasket slide was spun for 2 minutes at 2,000 rpm and cleaned with 70% EtOH before hybridization. Hybridization solution is prepared the same as the blocking solution, with the addition of 40nM Cy5 probe, which was the opposite allele of the unlabeled competitor. The protein microarray was hybridized using the Microarray Hybridization Chamber overnight at 4° in the dark on a nutating shaker. The protein microarray was washed with 4mL 1x Base buffer in a 4-well Nunc rectangular dish (ThermoFisher) for 2 minutes at 4° in the dark on an orbital shaker. Excess wash buffer was dabbed off the edges of the protein mircroarray, and it was spun in a micro slide box at 4° for 2 minutes at 2,000 rpm. The protein microarray was then imaged with GenePix 4000B scanner with 10um resolution at 635nm, 650 PMT Gain, and 100% power.

#### Data Analysis

GenePixPro was used to align the protein microarray list file, containing spot coordinates and protein names, to the protein microarray image file. Foreground and localized background intensities were calculated for each protein spot in GenePixPro. Signal intensity at each spot was quantified by dividing the median foreground intensity by the median local background intensity, and Z scores were calculated. Proteins with an average Z score above 4 on both protein microarray replicates for a given allele are considered positive. A protein is considered to bind both alleles of a SNP if the average Z score of both alleles is above 4 and the difference between the allele Z scores is less than 2. A protein is considered to bind both alleles of a SNP, but show preference for one allele if the average Z score is above 4, but the difference between allele Z scores is greater than 2. Otherwise, if only one allele exceeds the threshold of 4 the binding is considered allele specific.

### Circular Chromatin Conformation Capture with Next-Gen Sequencing (4C-seq)

#### Template preparation

4C-seq assays were performed as recommended by Splinter, *et al*. [45] with minor adjustments, including the reduction of centrifugation from 8346g to 3300g during sample purification.

The templates are a result of two digestion and ligation steps. The primary restriction enzyme digests crosslinked DNA, and the first ligation captures interacting DNA fragments. After the crosslinking is reversed, the secondary restriction enzyme and subsequent ligation are utilized to create shorter loops of interacting DNA that will be amplifiable to generate the sequencing libraries. Due to the nature of this experimental design, the resolution of interacting DNA fragments is limited to the size of fragments created by the primary restriction enzyme.

#### Illumina Sequencing

Primers for amplification of the 4C-seq library were designed as close as possible to restriction enzyme sites in the viewpoint fragment (*CACNA1C* or *CCAT* promoter), with tails that include barcodes for sample deconvolution and the Illumina TruSeq adapters. PCR and library purification were performed as recommended by Splinter, *et al*. [45]. Eight to 12 libraries were pooled in equal molarity for single end, 150 cycle sequencing on a MiSeq (Illumina).

#### Experimental design

To safeguard against false negatives due to cell type specific interactions we used two human cell lines: SK-N-SH neuroblastoma cells and the human embryonic kidney derived line HEK293. In follow up of positive results on SK-N-SH cells from the *CACNA1C* promoter viewpoint, we used 3 different primary restriction enzymes, so that the amplicons involved in each experiment would be different. We further performed each experiment in duplicate. We then applied quality controls requiring that we acquire at least one million reads from each experiment, that more than 80% of them originate from the viewpoint, and that more than a quarter can be mapped uniquely back to the human genome. This resulted in discarding some technical replicates. The benchmarks for the "passing" experiments are shown in S3 Table. When more than one technical replicate is available, we report results from that with better read count benchmarks, although the results do not differ significantly.

#### Data Analysis

The resulting “.fastq” files were processed through our 4C-seq analysis pipeline, which utilizes Unix shell scripts, Perl scripts, and R scripts. First, reads are separated and extracted to separate files by barcode (100% match required). Each barcode represents one experiment. Each file is then processed separately in the following steps. First, the sequences that match the sequence between the viewpoint primer and the primary restriction enzyme cut site are extracted, allowing for no more than two mismatches. This viewpoint sequence is removed so the resulting fragments now start at the ligation point of linked fragments. Next, any of these fragments containing secondary restriction sites are truncated at that site, as these would be hybrid fragments resulting from multiple fragment ligations. Next, the resulting fragments are aligned to the human genome (hg19) using Bowtie2 [46]. The reads mapping to the chromosome of interest (chromosome 12 for *CACNA1C*) are extracted and their start nucleotide position is noted. The start positions that map to primary enzyme restriction sites are counted, these counts representing the ligation events.

Many factors must be accounted for in order to quantitatively evaluate the frequency of DNA fragments interacting with the viewpoint. There are two types of fragments captured by the 4C template preparation, those that start at a primary site and end at a secondary site and those that have no secondary site but only primary sites on both ends. The latter, that we call “blind,” have an amplification disadvantage, as any potential amplicon would need to include a primary-to-secondary fragment in order to circularize and be amplified. The amplification efficiency also depends on fragment size, GC content, and distance from the viewpoint. While there is a strong negative correlation between counts and the distance from the viewpoint, the relationship is exponential at small distances. To correct for these factors, we generate a file that includes this information, as well as the squared fragment length and the squared distance from viewpoint for every one of the possible fragments at the region of interest, in this case 1Mb up and downstream from the viewpoint.

This file is then used as input to R to calculate a residual for the counts at each site through a generalized linear model. The residual counts corresponding to each side of each primary fragment are then merged to one value, the maximum of the two. We choose to do this (instead of using for example the average) because there are many potential causes for an amplicon to fail to generate mappable products or to amplify (e.g too long, too short, possible interference of binding proteins), even if it does partake in interactions. The rationale is that if one side shows a strong result, this is sufficient to suggest interactions. The resulting file, which now contains information on count residuals for each primary fragment, is passed again to R to calculate Z scores and p-values. Occasional very high counts, presumably from the strongest interactions, create a highly skewed distribution, which can lead to deflation of signal. To overcome this, the highest count fragments are iteratively removed and p-values recalculated. Finally, the resulting p-values are -log_10_ transformed and reported in a “.bed” format file, which can be loaded into the UCSC genome browser and visualized. To improve visibility, the -log(p-values) in this file are truncated to a max of 20. The analysis pipeline also uses R to graph the residual counts across the region to ensure that the effect of distance is corrected and create QQ plots of the residuals to confirm that their distribution after all the adjustments is near normal with only relatively few high values as expected for true interactions.

## Supporting Information

S1 Fig Schematic representation of the qPCR primer design. Primer design for the three classes of *CACNA1C* transcripts as named by Tang, *et al*. (WT, B, and D) [21]. Thick grey boxes represent exons, which are numbered above. Solid arrows represent primers and those designed across introns are connected by dashed lines; primers span the exon junction in order to uniquely amplify a single class of cDNA transcripts.
(TIFF)

S2 Fig Relative firefly luciferase activity for DLR constructs. Constructs transfected in HEK293 cells shown on the top, and SK-N-SH cells shown on the bottom. Firefly/Renilla ratio is normalized to 1.0 for the control construct. The SNP name(s) within each construct is listed below the bars. Each bar represent the average of four independent construct DNA extractions and error bars represent standard error. Red bars correspond to risk alleles and blue bars to the non-risk alleles. T-test based p-values are shown above the pair only when there is a significant difference between alleles.
(TIF)

S3 Fig Replications of a subset of DLR constructs transfected in HEK293 cells. A subset of data from Fig 2. is shown again in the top panel. Relative firefly luciferase activity is shown as an average of four constructs for each allele. Error bars represent standard error. Non-risk allele is shown in blue, risk allele is in red. Significant differences between the two alleles of a construct are indicated as p-values above the pair.
(TIFF)

S4 Fig EMSAs for all 16 SNPs tested. Reaction components present are indicated by a “+” above each lane. Controls A and B are positive controls from our lab. Controls C is the non-risk allele (G) of rs4765905.
(PDF)

S5 Fig 4C-seq results in HEK293 cells. The viewpoint is the *CACNA1C* promoter, as indicated by the arrow. Peaks in the two tracks indicate–log p-values of regions that interact with the viewpoint. The defined regions, *CACNA1C* PROMOTER, PSY-SNPs, and REGION A are the same as shown in Fig 4.
(TIF)

S1 Table Position and alleles of all 16 SNPs in the LD block tagged by rs1006737. These variants are in high LD with the disease-associated SNP rs1006737, and were therefore included in DLR constructs and as probes for both EMSAs and protein microarrays.
(XLSX)

S2 Table Protein microarray results for all 16 SNPs and 3 control oligos examined. “NR” indicates protein binds only to the non-risk allele, “R” indicates the protein binds only the risk allele, “B” indicates protein binds both alleles, “+” indicated binding to control oligo, “n/a” indicates that the protein was not included on the previous version of the array where 460 DNA oligos were tested, blank cells indicate no binding. The bolded proteins bind at least 3 of the 16 SNPs, but were not present on the previous version of the array, so we do not have binding frequency data.
(XLSX)

S3 Table Summary of sequencing metrics for 4C-seq assay. Viewpoint indicates which promoter, *CACNA1C* or *CCAT*, was used. The “Reads” column indicates the total number of reads starting with the barcode unique to that experiment, followed by the percent of reads mapping to the viewpoint sequence, and the percent of reads that continue beyond the viewpoint to an interacting fragment on chromosome 12, where *CACNA1C* and *CCAT* are located.
(XLSX)
