# Supplementary figures and images for: Functional Characterization of Schizophrenia-Associated Variation in CACNA1C
Source: PLoS One. 2016 Jun 8;11(6):e0157086. doi: 10.1371/journal.pone.0157086 (PMC4898738; doi:10.1371/journal.pone.0157086)

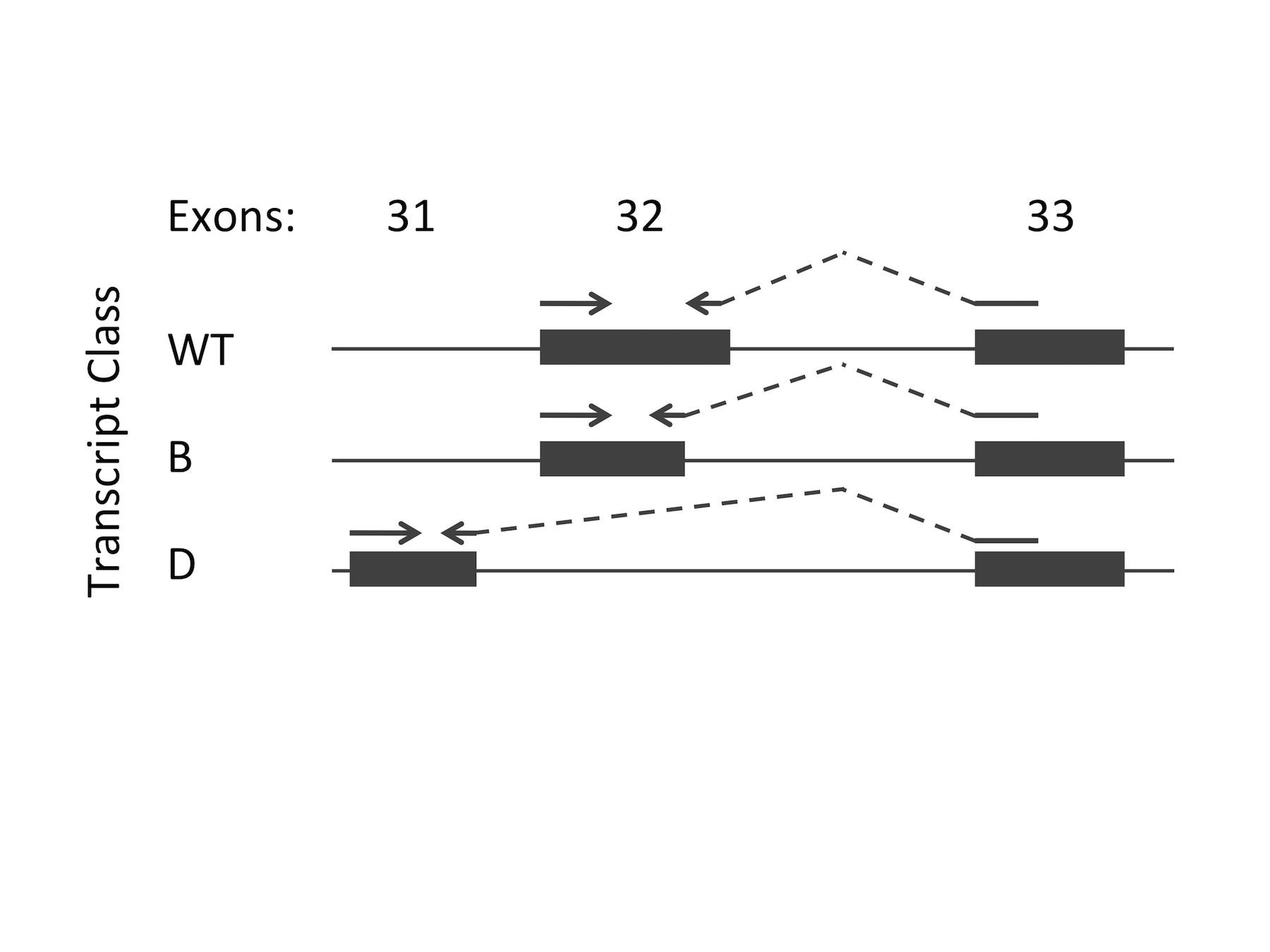

Supplement: S1 Fig — Primer design for the three classes of CACNA1C transcripts as named by Tang, et al. (WT, B, and D) [21]. Thick grey boxes represent exons, which are numbered above. Solid arrows represent primers and those designed across introns are connected by dashed lines; primers span the exon junction in order to uniquely amplify a single class of cDNA transcripts. (TIFF) [file pone.0157086.s001.tiff]

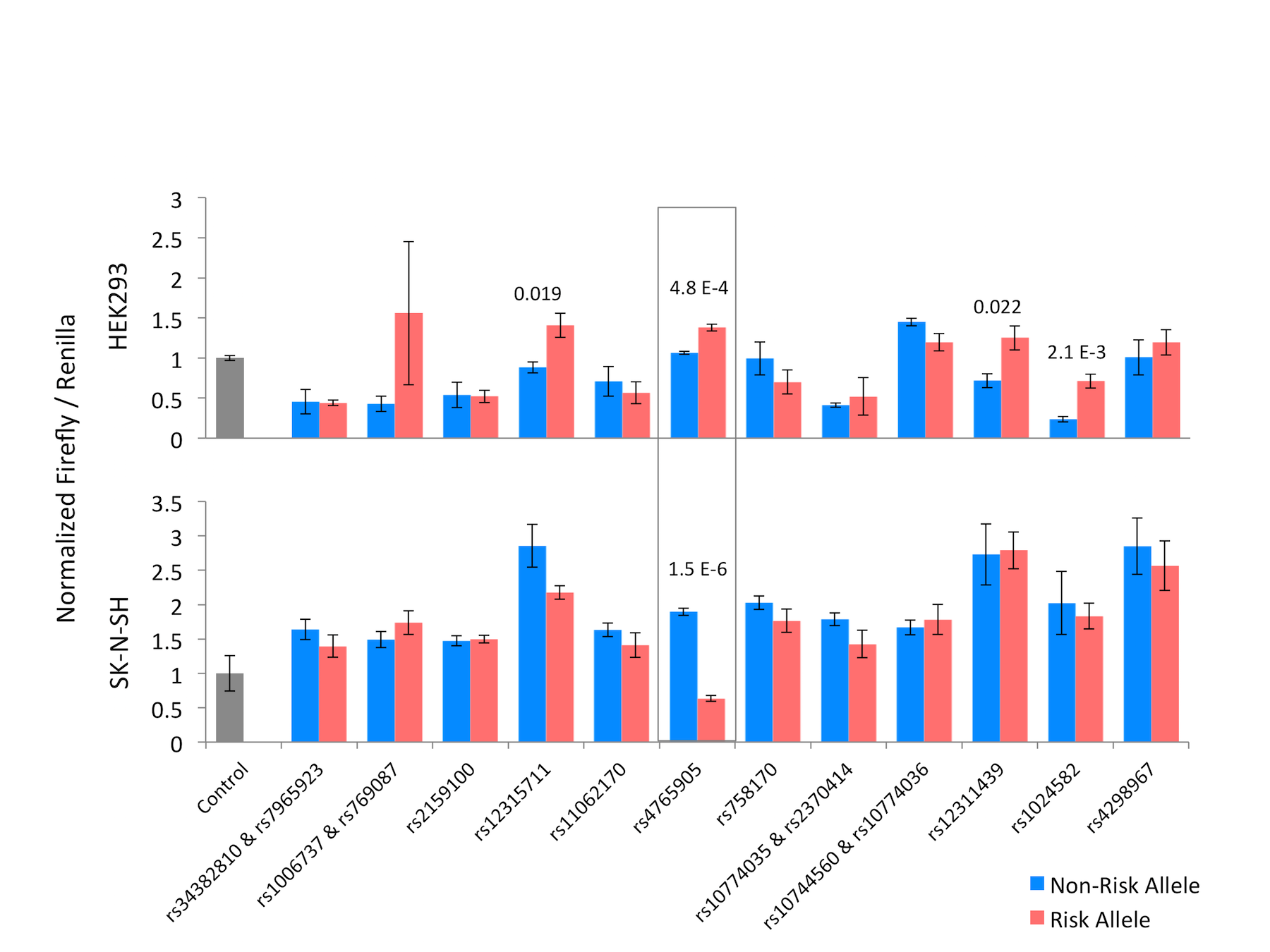

Supplement: S2 Fig — Constructs transfected in HEK293 cells shown on the top, and SK-N-SH cells shown on the bottom. Firefly/Renilla ratio is normalized to 1.0 for the control construct. The SNP name(s) within each construct is listed below the bars. Each bar represent the average of four independent construct DNA extractions and error bars represent standard error. Red bars correspond to risk alleles and blue bars to the non-risk alleles. T-test based p-values are shown above the pair only when there is a significant difference between alleles. (TIF) [file pone.0157086.s002.tif]

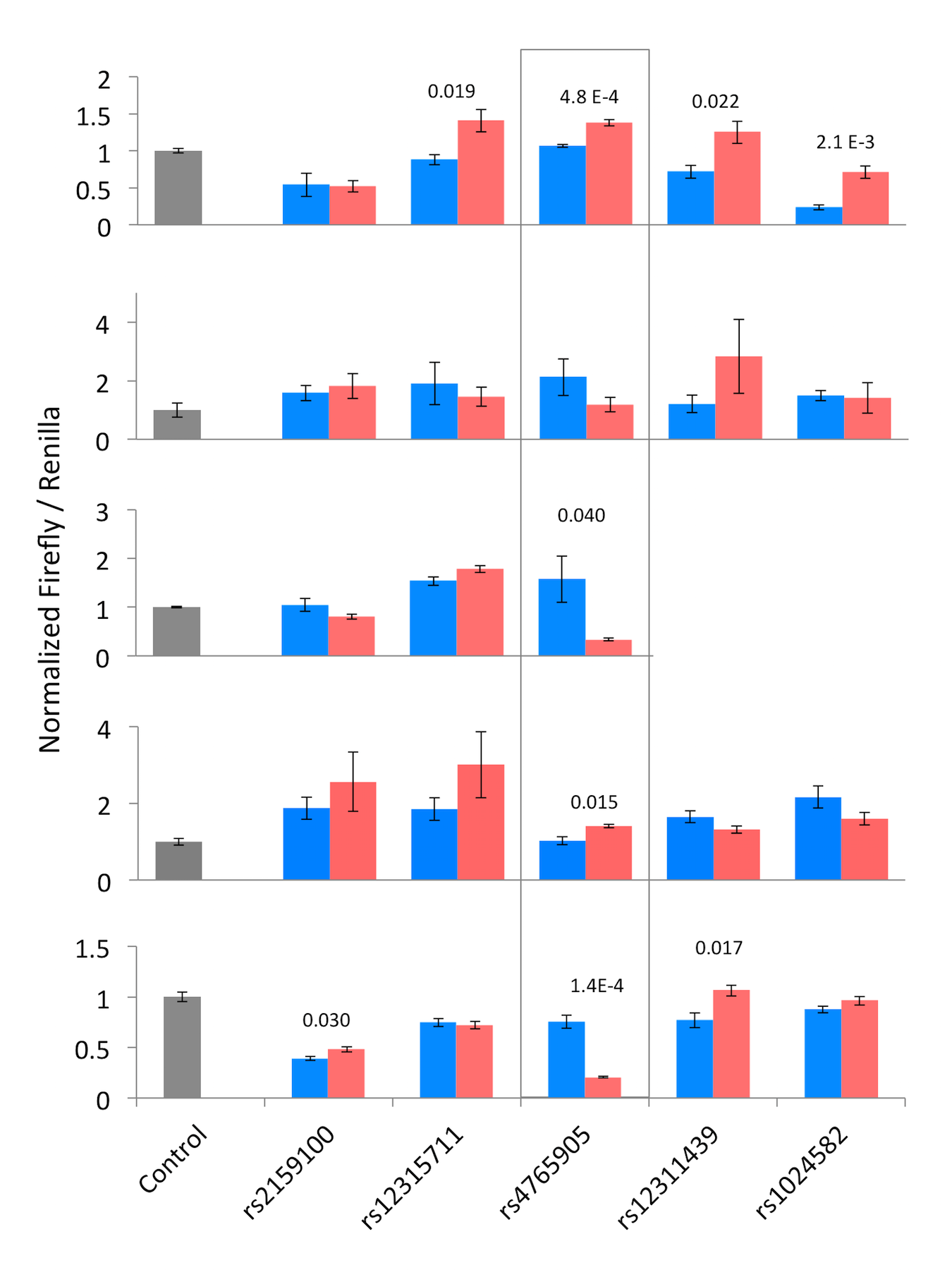

Supplement: S3 Fig — A subset of data from Fig 2. is shown again in the top panel. Relative firefly luciferase activity is shown as an average of four constructs for each allele. Error bars represent standard error. Non-risk allele is shown in blue, risk allele is in red. Significant differences between the two alleles of a construct are indicated as p-values above the pair. (TIFF) [file pone.0157086.s003.tiff]

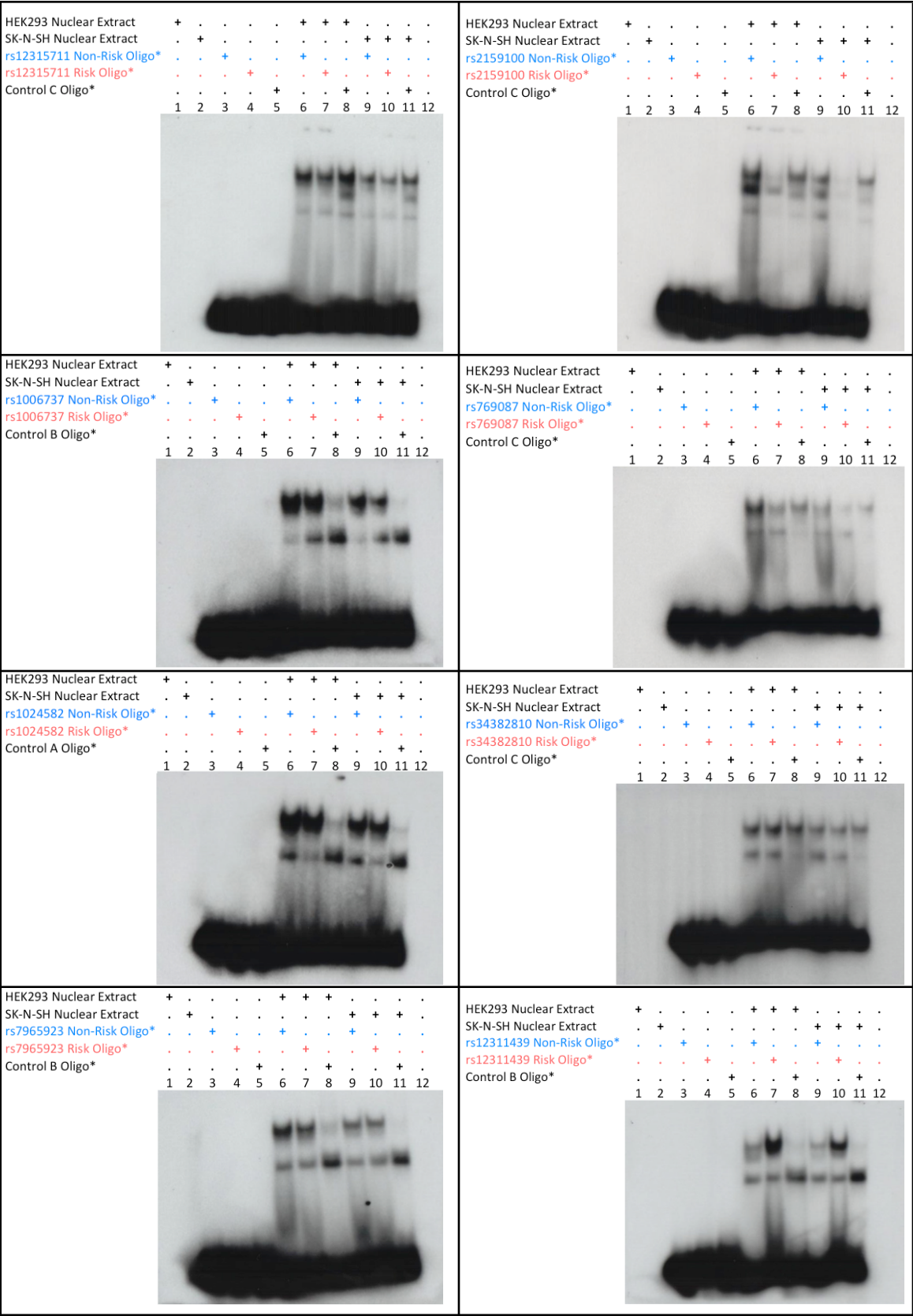

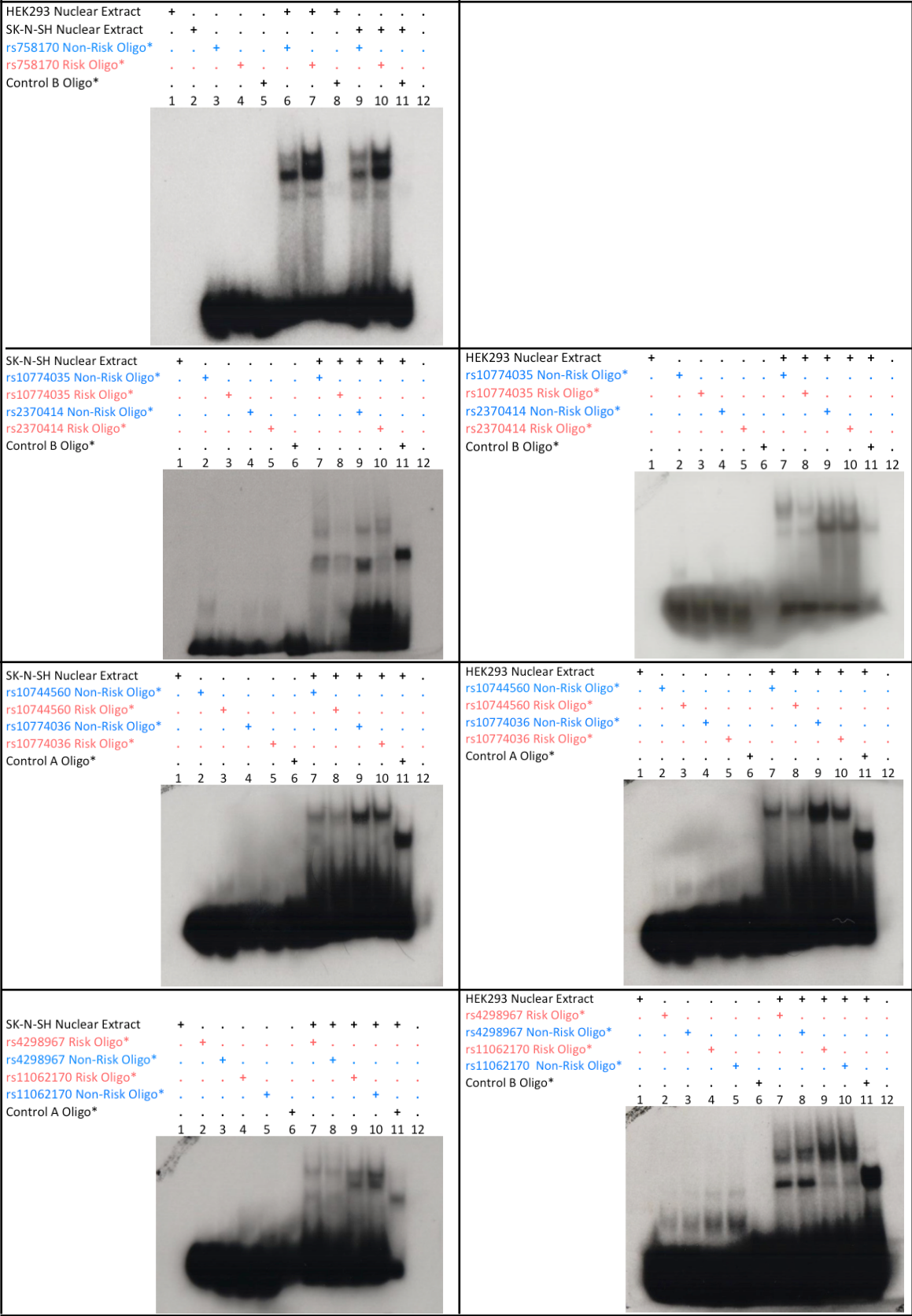

Supplement: S4 Fig — Reaction components present are indicated by a “+” above each lane. Controls A and B are positive controls from our lab. Controls C is the non-risk allele (G) of rs4765905. (PDF) [file pone.0157086.s004.pdf]

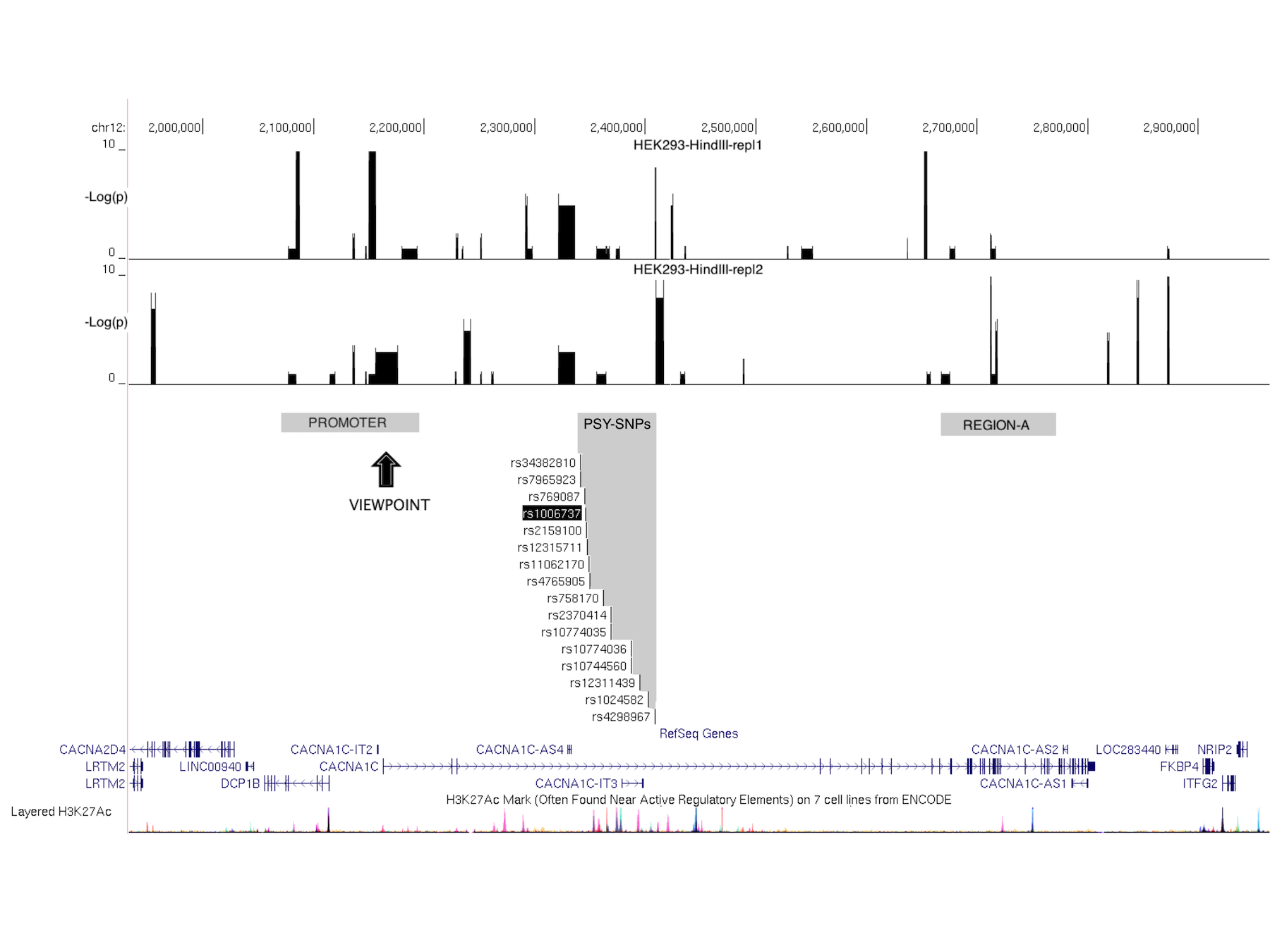

Supplement: S5 Fig — The viewpoint is the CACNA1C promoter, as indicated by the arrow. Peaks in the two tracks indicate–log p-values of regions that interact with the viewpoint. The defined regions, CACNA1C PROMOTER, PSY-SNPs, and REGION A are the same as shown in Fig 4. (TIF) [file pone.0157086.s005.tif]
